# Pushing the boundaries of resistance: insights from *Brachypodium*-rust interactions

**DOI:** 10.3389/fpls.2015.00558

**Published:** 2015-07-30

**Authors:** Melania Figueroa, Claudia V. Castell-Miller, Feng Li, Scot H. Hulbert, James M. Bradeen

**Affiliations:** ^1^Department of Plant Pathology, University of Minnesota, St. Paul, MN, USA; ^2^Stakman-Borlaug Center for Sustainable Plant Health, University of Minnesota, St. Paul, MN, USA; ^3^Department of Plant Pathology, Washington State University, Pullman, WA, USA; ^4^Department of Crop and Soil Sciences, Washington State University, Pullman, WA, USA

**Keywords:** *Brachypodium*, rust fungi, *Puccinia*, plant immunity, non-host resistance

## Abstract

The implications of global population growth urge transformation of current food and bioenergy production systems to sustainability. Members of the family Poaceae are of particular importance both in food security and for their applications as biofuel substrates. For centuries, rust fungi have threatened the production of valuable crops such as wheat, barley, oat, and other small grains; similarly, biofuel crops can also be susceptible to these pathogens. Emerging rust pathogenic races with increased virulence and recurrent rust epidemics around the world point out the vulnerability of monocultures. Basic research in plant immunity, especially in model plants, can make contributions to understanding plant resistance mechanisms and improve disease management strategies. The development of the grass *Brachypodium distachyon* as a genetically tractable model for monocots, especially temperate cereals and grasses, offers the possibility to overcome the experimental challenges presented by the genetic and genomic complexities of economically valuable crop plants. The numerous resources and tools available in *Brachypodium* have opened new doors to investigate the underlying molecular and genetic bases of plant–microbe interactions in grasses and evidence demonstrating the applicability and advantages of working with *B. distachyon* is increasing. Importantly, several interactions between *B. distachyon* and devastating plant pathogens, such rust fungi, have been examined in the context of non-host resistance. Here, we discuss the use of *B. distachyon* in these various pathosystems. Exploiting *B. distachyon* to understand the mechanisms underpinning disease resistance to non-adapted rust fungi may provide effective and durable approaches to fend off these pathogens. The close phylogenetic relationship among *Brachypodium* spp. and grasses with industrial and agronomic value support harnessing this model plant to improve cropping systems and encourage its use in translational research.

## Introduction

Cereals, which are classified within the grass family Poaceae, also known as Gramineae, are essential worldwide commodities (Chapman, 1996). The importance of these plant species ranges from economic to ecological, and stems from their multiple applications in industry, food production, livestock feed, generation of renewable and sustainable energy among others. The critical role of cereals in human nutrition is highlighted by the fact that wheat, corn, and rice provide nearly two thirds of the global caloric intake (Tilman et al., 2011). Other crops, like oat and millet, exemplify the extraordinary nutritional value that cereals can provide (Bewley et al., 2012; Shukla and Srivastava, 2014). In a different context, the cultivation of grasses such as switchgrass, sweet sorghum, *Miscanthus*, and especially sugarcane and corn, are the main basis of biofuel production (Somerville, 2007) increasing the value of these plant species.

According to predictions of worldwide population growth, agricultural production must increase approximately twofold to meet the dietary needs of nine billion people in the year 2050 (US Census Bureau, 2015; Tilman et al., 2011). In addition to this, crop production systems need to consider a dietary bias toward higher consumption of meat and dairy, and the demands of increasing biomass to support biofuel production (Ray et al., 2013). Increasing crop yields and boosting agricultural production are among the greatest challenges humanity faces, as research suggests current crop yield trends are not on track to reach our target food production (Ray et al., 2013). Unpredictable and recurrent epidemics caused by plant pathogens are factors that have hampered productivity of crops since the birth of agriculture about 10,000 years ago (Oerke, 2006). The mid 1800s Irish potato famine caused by the oomycete *Phytophthora infestans* (Woodham-Smith, 1991) and other similar epidemics demonstrate the influence of plant diseases in history and society (Schumann, 1991). Currently, pre-harvest plant disease causes an approximately 15% loss of global crop production (Popp and Hantos, 2011).

The global production of small grains is severely affected by rust fungi (Maier et al., 2003). Some of these economically important pathogens include members of the genus *Puccinia* such as *P. graminis*, which affects production of wheat (*Triticum aestivum* and *T. durum*), barley (*Hordeum vulgare*) and oat (*Avena sativa*), *P. triticina*, the causal agent of wheat leaf rust, *P. striiformis*, which causes stripe rust on wheat, and *P. coronata*, the causal agent of oat crown rust (Cummins, 1971). In fact, the recurrent outbreaks of *P. coronata* f. sp. *avenae* and the rapid emergence of hypervirulent races of the wheat stem rust fungus, *P. graminis* f. sp. *tritici* (i.e., TTKSK) are two examples that illustrate the potential devastating economic effects of rust fungi on crops around the globe (Pretorius et al., 2000; Leonard and Martinelli, 2005; Singh et al., 2006; Park, 2008; Pennisi, 2010). This review discusses the biological attributes of rust fungi, and how those characteristics can contribute to expand our knowledge in plant immunity. We focus on the development of *Brachypodium*-rust pathosystems, which are important at two fronts, first helping us to understand the principles of non-host resistance (NHR) and second holding promise to enhance disease resistance engineering technologies.

## The Two Faces of Rust Fungi—Dangerous Pathogens and Good Models

Rust fungi exhibit complex lifecycles with both sexual and asexual (clonal) cycles of reproduction. In the case of the previously listed *Puccinia* species, sexual reproduction is completed on an alternate host as these pathogens undergo a heteroecious life cycle (Al-Kherb et al., 1987; Jin et al., 2010). In contrast, other rust fungi known to be autoecious, like the causal agent of flax rust *Melampsora lini*, depend solely on one host to complete both asexual and sexual reproduction (Lawrence et al., 2007). An interesting biological characteristic of rust fungi is that they generally show high levels of host specificity, although there is variability in host range exhibited by these pathogens. For instance, species like *P. triticina* infect a limited number of plant species (Roelfs et al., 1992), while others like *P. graminis* and *P. coronata* have a host range in the order of hundreds of related species (Anikster, 1984; Wahl et al., 1984; Leonard and Szabo, 2005). However, in these cases, the rust species can generally be classified into different physiological variants known as formae speciales (ff. spp.), each of them having a much more restricted host range (Anikster, 1984). For example, *P. graminis* f. sp. *avenae* affects only oat production, while *P. graminis* f. sp. *tritici* targets barley and wheat (Leonard and Szabo, 2005). The investigation of the evolutionary and physiological factors determining host range and specificity of rust species and formae speciales is of interest as those factors could provide insights into the mechanisms that drive pathogen jumps from one host species to another and the emergence of new diseases (Bettgenhaeuser et al., 2014). Unraveling the riddles of sexual reproduction in rust fungi, the biological contribution of alternate hosts in introducing genetic variation, particularly virulence alterations, can also help understand the rise of new pathogens or pathotypes.

Rust fungi have co-evolved with their respective host(s) for millions of years, a process that has resulted in the adoption of complex and effective infection strategies involving specialized cell types and morphological structures (Harder, 1984; Harder and Chong, 1984; Wahl et al., 1984). Such complexity is manifested by the production of up to five type of spores: basidiospores, pycniospores, aeciospores, urediniospores, and teliospores. However, most research in developmental biology of rust fungi has been directed to the infection mediated by urediniospores as they are responsible for multiple infection cycles in the gramineous host with agronomic value (Leonard and Szabo, 2005). *P. graminis* f. sp. *tritici* is one of the best characterized pathogenic rust fungi and has been widely used to describe the morphological differentiation processes associated with rust diseases of cereals. The elaborate developmental program followed by rust fungi is initiated by the recognition of the host environment, where chemical, thigmotropic and topographical characteristics of the leaf surface prompt hyphal growth and orientation (Hoch et al., 1987; Heath, 1997; Brand and Gow, 2012). Moisture on the leaf surface induces the germination of urediniospores, a process that is followed by the elongation of a germ tube and location of a stoma, which serves as a cue to form an appressorium and gain access to internal cavities of the plant (Staples and Macko, 2004; Leonard and Szabo, 2005). The course of infection continues with the differentiation of a substomatal vesicle and the emergence of a primary hypha that quickly extends in the plant mesophyll space. During more advanced stages of infection, feeding structures, referred to as haustoria, penetrate the walls of living plant cells, and invaginate the host plasma membrane forming an intimate association with the plant (Harder and Chong, 1984; Staples and Macko, 2004). Finally, the colonization leads to sporulation and the generation of uredial pustules erupting through the epidermis of the leaf (Leonard and Szabo, 2005). Management of diseases caused by rust fungi is often a difficult task as the pathogen grows asymptomatically during colonization and disease does not manifest until the fungus is undergoing sporulation (Leonard and Szabo, 2005). In this context, furthering our understanding of biochemical and molecular events particularly during early stages of infection should be regarded as a research priority.

The effectiveness of introducing disease resistance into crops to counteract the negative effect of rust fungi was established in the very early days of plant breeding with the identification of a resistance gene effective against *P. striiformis* (Biffen, 1907). Early studies in the flax-*M*. *lini* pathosystem played an important role in establishing the concepts of plant immunity, especially Flor’s research leading to the “gene-for-gene” model, and demonstrate the usefulness of rust fungi to uncover the most fundamental principles in plant pathology (Flor, 1971; Lawrence et al., 2007; Ellis et al., 2014). It is now evident that the genetically determined plant immune system constitutes the underlying basis of breeding crop plants for pathogen resistance and dissecting the factors that govern the onset of rust disease can bring innovation to crop protection programs (Dodds and Rathjen, 2010). The distinctive morphological features that rust fungi adopt during infection, as well as the extended period of time that these pathogens spend in contact with their host, enable ideal experimental systems to elucidate how the fungus copes with the plant defenses and resolve molecular changes in relationship to infection stages at a high-resolution.

## The Plant Immune System and the Pursuit of Crop Health

In nature plants are constantly threatened by different microorganisms; however, the development of disease as an outcome of those interactions is rather uncommon (Dangl and Jones, 2001; Lipka et al., 2010). Whether a plant is suitable as a host to a particular would-be pathogen is partly conferred by a battery of physiological and biochemical immune responses that result in a wide array of phenotypes ranging from complete immunity (incompatibility) to full susceptibility (compatibility). Plant disease resistance has been classified historically as either host resistance, describing resistance that is specific to a plant cultivar, variety or accession to a pathogen that is able to infect other host genotypes of that species (Flor, 1971; Jones and Dangl, 2006; Dodds and Rathjen, 2010), or NHR, referring to a type of resistance that is present across all genotypes of a plant species and prevents infection by any genetic variants (i.e., formae speciales, races, isolates) of a given non-adapted pathogen (Heath, 1981, 2000; Mysore and Ryu, 2004; Bettgenhaeuser et al., 2014). Considering that most of the naturally occurring disease resistance is attributed to NHR, this type of plant immunity holds great promise to agricultural practices as means to provide durable and broad-spectrum resistance (Mysore and Ryu, 2004; Ellis, 2006; Schulze-Lefert and Panstruga, 2011). Our present understanding of the mechanisms underlying NHR is much less extensive than the accumulated body of knowledge that conveys the molecular basis of host resistance. Recent evidence suggests that components mediating host resistance are likely important determinant factors in NHR, and thus both types of resistance may share some of the same inducible responses (Huitema et al., 2003; Mysore and Ryu, 2004; Douchkov et al., 2014; Lee et al., 2014). Consequently, the distinction between host and NHR may not truly reflect mechanistic differences among the two types of resistance, as both phenomena may exploit the same means of pathogen perception and defense.

Microbial recognition follows a two-tier system, enabling the plant to mount a defense response that may or may not be effective depending on the physiological characteristics of the would-be pathogen. First, the plant is equipped to recognize conserved pathogen-associated molecular patterns (PAMPs) such as the fungal cell wall component chitin (Jones and Dangl, 2006; Dodds and Rathjen, 2010). This phenomenon, known as PAMP-triggered immunity (PTI), is thought to be particularly relevant in NHR as it effectively prevents infection by non-adapted pathogens (Schulze-Lefert and Panstruga, 2011). PTI operates via plant cell surface-associated receptors (pattern recognition receptors, PRRs) that activate various basal defense responses such as callose deposition, production of reactive oxygen species, activation of pathogenesis-related proteins, etc. (Monaghan and Zipfel, 2012; Fu and Dong, 2013). The second tier of plant defense, known as effector-triggered immunity (ETI), exploits the backbone of many pathogens’ virulence strategies. Pathogens secrete proteins and small molecules, known as effectors, which among other functions can suppress or inhibit PTI-associated defense responses to allow infection to proceed (Hogenhout et al., 2009; Koeck et al., 2011). During ETI, the plants recognize effectors via intracellular receptor proteins of the nucleotide binding leucine rich repeat (NB-LRR) class, turning on complex cellular responses in order to restrict pathogen growth. These receptors are encoded by resistance (*R*) genes, and, in general, embody the traditional “gene-for-gene” concept initially described in the flax rust pathosystem (Flor, 1971; Jones and Dangl, 2006; Dodds and Rathjen, 2010). The recognition of effectors is typically associated with a rapid and localized cell death reaction at the site of infection, known as the hypersensitive response (HR); however, HR-like cell death can also occur during PTI (Khatib et al., 2004). The ETI concept is generally considered to encompass recognition of effectors delivered/translocated into the host cell; however, some effectors accumulate in apoplastic space and exert their function extracellularly (Hogenhout et al., 2009). Some of these apoplastic effectors are recognized by plasma membrane-associated receptors in the plant via a mechanism that differs from ETI and PTI (Stotz et al., 2014). This type of resistance, named effector-triggered defense (ETD), is commonly associated with apoplastic fungal leaf pathogens and can be manifested as slow developing cell death (Stotz et al., 2014). Regardless of the terminology and mechanistic differences between ETD and ETI, ETD should be regarded as part of the second tier of microbial recognition as its fundamental principle is still recognition of effector molecules. Successful infection by rust fungi requires haustorium-mediated delivery and translocation of effectors into the invaded plant cells (Catanzariti et al., 2006, 2007; Panstruga and Dodds, 2009; Garnica et al., 2014), and while the secretion of effectors via other structures has not been demonstrated it is possible that those events take place and are relevant to the rust pathogen–plant interaction outcomes. The presence of apoplastic effectors produced by rust fungi has not been demonstrated either, but it seems reasonable to think that this class of effectors could be important during early stages of rust infection, prior the differentiation of the first haustorium. Barley-*P. graminis* interactions mediated by the *Rpg1* gene clearly begin before haustorial formation since the RPG1 protein is phosphorylated within 5 min of spores from avirulent pathotypes contacting the leaf surface (Nirmala et al., 2010). According to these findings, two effector proteins required for the phosphorylation and subsequent degradation of RPG1 are present on the spore surface and probably other fungal structures (Nirmala et al., 2011).

In addition to immune system-induced resistance, genetic protection from disease can occur through the action of resistance genes that have other effects. These genes often provide broad-spectrum resistance against multiple pathogens, and, although frequently referred to as resistance genes, do not necessarily encode receptor-like proteins like NB-LRR proteins. An example of this type of resistance in wheat is the response conditioned by the gene *Lr34*, which encodes a putative adenosine triphosphate-binding cassette (ABC) transporter protein, and confers partial resistance against leaf, stem and stripe rust fungi, as well as barley yellow dwarf virus and powdery mildew (Dyck et al., 1966; Singh, 1993; Krattinger et al., 2009; Risk et al., 2013). In summary, this broad array of defense mechanisms represents targets that may be exploited in various strategies to prevent and minimize crop losses.

## *Brachypodium distachyon*: A Model System that Defies Rust Diseases

Throughout research history, the use of experimental model organisms such as *Escherichia coli*, *Saccharomyces cerevisiae*, *Neurospora crassa*, and *Caenorhabditis elegans* have accelerated scientific discovery and led to significant breakthroughs (Miklos and Rubin, 1996; Guarente and Kenyon, 2000; Davis and Perkins, 2002; Replansky et al., 2008). Research in wheat, oat, and other monocots often faces long-standing challenges associated with the ploidy of the plants and the large size of their genomes (Chawade et al., 2010; Mayer et al., 2014). The use of representative model species can help circumvent those problems and accelerate the pace of research. In addition, leveraging basic knowledge from model organisms for applied purposes, such as disease resistance, can be extremely advantageous to crop improvement. Dicotyledonous species such as *Arabidopsis thaliana* (thale cress or mouse-ear cress) and *Solanum lycopersicum* (tomato) have served as excellent models to elucidate the plant immune system, understand the basis of plant–microbe interactions and have made significant contributions to crop improvement (Piquerez et al., 2014). However, dicotyledonous and monocotyledonous plants diverged at least 140–150 million years ago (Chaw et al., 2004; Davies et al., 2004; Anderson et al., 2005) and the substantial phylogenetic distance between cereals and dicots can often hinder downstream applications of knowledge gained from dicot models to cereal research and breeding. Thus, there is need for developing appropriate systems to study monocotyledonous plants. Acknowledging this necessity and the role of cereals in food security, rice (*Oryza sativa* L.) was launched as a genetic and genomic model several years ago (Goff et al., 2002; Project, 2005). These efforts facilitated advances in comparative genomics, and demonstrated that characterization of the genic content and landscape of one grass species could be informative and applicable to other related species (Gale and Devos, 1998; Phillips and Freeling, 1998). Rice also turned out to be a useful model to study plant immunity and the existing genomic resources and tools in this species are highly valuable to plant scientists and breeders (Phillips and Freeling, 1998; Chen and Ronald, 2011; Chen et al., 2013). Nevertheless, basic biological and physiological differences between *Oryza* spp. and temperate grasses can hinder rice’s utility to inform on certain processes, like rust resistance, in cool-season crops (Phillips and Freeling, 1998; Draper et al., 2001; Vogel and Bragg, 2009; Chen et al., 2013). Disease resistance in rice to important cereal pathogens is not always genetically tractable. While rice acts as a non-host to all rust species, the variation in NHR phenotypes across cultivars can be subtle and often influenced by environmental factors; limiting the value of rice in rust research (Ayliffe et al., 2011, 2013).

To tackle some of these challenges, the small wild grass *Brachypodium distachyon* (Pooideae subfamily), also known as purple false brome, was developed as an experimental model to investigate temperate grass biology (Draper et al., 2001; Vogel and Bragg, 2009; Brkljacic et al., 2011; Mur et al., 2011). *B. distachyon* diverged from the wheat phylogenetic lineage approximately 35–40 million years ago indicating it is more closely related to Triticeae than rice (Bossolini et al., 2007). As a species, *B. distachyon* has no agronomic value; however, it possesses biological attributes advantageous for its use as a model system (Draper et al., 2001; Vogel and Bragg, 2009; Mur et al., 2011). Similar to *A. thaliana*, *B. distachyon* exhibits a small plant size, compact genome (∼270–300 Mb), short life cycle (2–3 months), genetic tractability, and minimal growth requirements. There are several species within the genus *Brachypodium* and their taxonomic classification has been recently resolved (Catalán et al., 2012; López-Alvarez et al., 2012). *B. distachyon* and *B. stacei* include diploid plants with 5 and 10 pairs of chromosomes, respectively; whereas *B. hybridum* is an allotetraploid species that resulted from a hybridization event between *B. distachyon* and *B. stacei* (López-Alvarez et al., 2012). The different ploidies within the genus offers opportunity to conduct evolutionary, gene inheritance and expression studies in a system that is easier to handle than related crops like wheat and oat. The embracement of *Brachypodium* as model has resulted in a wealth of genetic, genomic, and experimental assets that set the stage to answer many interesting questions in plant biology and pathology. Some of the established resources include large natural germplasm collections and several families of recombinant inbred lines available to the scientific community (Jenkins et al., 2003; Vogel et al., 2006a, 2009; Vogel and Hill, 2008; Scoles et al., 2009). In addition, there is access to bacterial artificial chromosome (BAC) and expressed sequence tag (EST) libraries (Vogel et al., 2006b; Huo et al., 2008), genetic and physical maps (Febrer et al., 2010), simple sequence repeat (SSR) microsatellite markers (Azhaguvel et al., 2009; Vogel et al., 2009; Garvin et al., 2010), a high quality Sanger-based reference genome assembly of *B. distachyon* inbred line Bd21 and deep sequencing data corresponding to additional genotypes^1^ (International Brachypodium Initiative, 2010; Gordon et al., 2013). The implementation of efficient *Agrobacterium tumefaciens*-mediated transformation methods allowed the creation of two large banks of T-DNA insertion mutants in *B. distachyon*^2^^,^^3^, which are also a great resource available to the community (Alves et al., 2009; Bragg et al., 2012; Thole et al., 2012).

From the perspective of research in plant immunity, *B. distachyon* is emerging as a suitable system to investigate plant–microbe interactions (Fitzgerald et al., 2015). In contrast to rice, *Brachypodium* species can act as a host to *Puccinia brachypodii*, an adapted pathogen of *B. sylvaticum*, thus better positioning this system to investigate various aspects of rust compatibility (Barbieri et al., 2011). Initial pathogenicity tests on various ecotypes of *B. distachyon* showed the potential of the species to investigate the molecular basis of resistance against pathogens like *Blumeria graminis*, *Magnaporthe oryzae*, and various rust fungi such as *P. hordei*, *P. triticina*, *P. striiformis* f. sp. *tritici* and *P. striiformis* f. sp. *hordei* (Draper et al., 2001; Routledge et al., 2004; Peraldi et al., 2011). Since these early reports, the number of studies examining the interaction of *B. distachyon* with pathogenic fungi, some of which affect food and biofuel crops, is growing. *B. distachyon* has been established as a pathosystem to study important grain diseases such as to *Fusarium* head blight, caused by *Fusarium graminearum* (Peraldi et al., 2011), *Rhizoctonia* root rot in wheat, caused by *Rhizoctonia solani* (Schneebeli et al., 2014), spot blotch and common root rot caused by *Cochliobolus sativus* (Zhong et al., 2014), Septoria tritici blotch disease, caused by *Zymoseptoria tritici* (O’Driscoll et al., 2015), take-all caused by *Gaeumannomyces graminis* (Sandoya and de Oliveira Buanafina, 2014), Stagonospora nodorum blotch and net blotch of barley caused by *Pyrenophora teres* (Falter and Voigt, 2014), as well as other grass diseases caused by *Sclerotinia homoeocarpa* and *Ophiosphaerella agrostis* (Sandoya and de Oliveira Buanafina, 2014).

**FIGURE 1:**
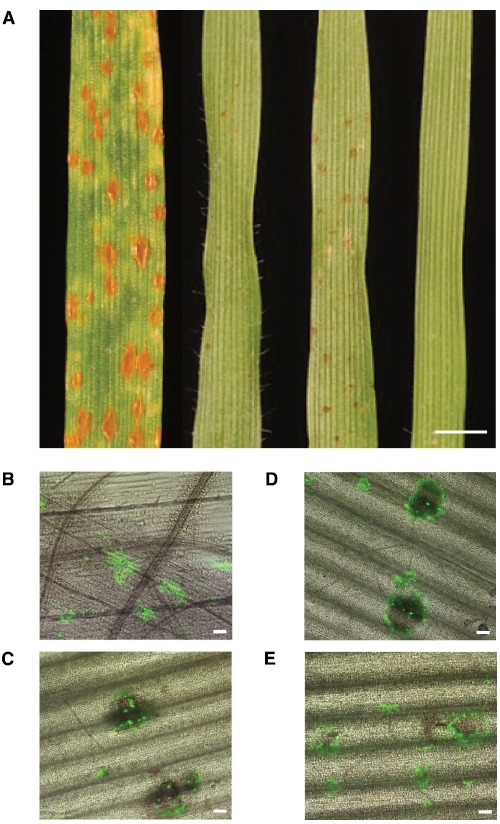
**Symptoms induced by ***P. graminis*** f. sp. ***tritici*** in wheat and ***B. distachyon***. (A)** Left to right, macroscopic symptoms of *P. graminis* f. sp. *tritici* (CRL 75-36-700-3) in susceptible *Triticum aestivum* cv. McNair 701, *B. distachyon* inbred lines Bd1-1, Bd2-3, Bd21 at 12 days after inoculation, scale = 2 mm. **(B–E)** Microscopic symptoms induced by *P. graminis* f. sp. *tritici* and various inbred lines of *B. distachyon* at 96 h post-inoculation. Inoculations were performed as previously reported (Figueroa et al., 2013). Fungal tissue was stained with wheat germ agglutinin conjugated to fluorescein isothiocyanate (WGA-FITC; Sigma-Aldrich, St. Louis, MO, USA) as previously described (Ayliffe et al., 2013). **(B)** Susceptible *Triticum aestivum* cv. McNair 701. **(C)** Inbred line Bd1-1. **(D)** Inbred line Bd2-3. **(E)** Inbred line Bd21. Images were generated by merging bright and fluorescence fields captured using an Olympus IX70 Inverted Fluorescence Microscope, scale = 100 μm.

**FIGURE 2:**
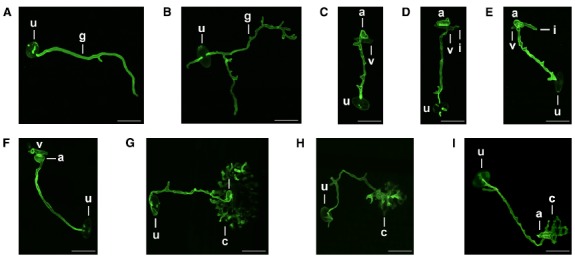
**Differentiation of morphological structures associated with the infection of ***P. graminis*** f. sp. ***tritici*** on ***B. distachyon***.** *P. graminis* f. sp. *tritici* (CRL 75-36-700-3) was used to inoculate various *B. distachyon* inbred lines following procedures previously reported (Figueroa et al., 2013). Fungal tissue was stained WGA-FITC as previously described (Ayliffe et al., 2013). **(A,B)** Germination of urediniospores (u) on the surface of leaves of *B. distachyon* inbred line Bd1-1, micrographs illustrate the different morphological features of germ tubes as they elongate to locate a stoma. **(C–E)** Differentiation of an appressorium (a) and substomatal vesicle (v) in *B. distachyon* inbred lines Bd1-1, Bd2-3, Bd21, respectively, at 24 hpi (10 h of light exposure). Notice differences in the growth of primary infection hyphae (i) as it emerges from the substomatal vesicle. **(F)** Example of an infection site in *B. distachyon* inbred line Bd1-1 that failed to continue growth after forming the appressorium and substomatal vesicle. Image was capture at 96 hpi. **(G–I)** Presence of rust fungal colonies in *B. distachyon* inbred lines Bd1-1, Bd2-3, Bd21, respectively, at 96 hpi. Images were captured using epifluorescence in a Nikon A1 Spectral Confocal Microscope, scale = 50 μm.

The role of *Brachypodium* spp. as a non-host to formae speciales of rust fungi, notably *P. graminis*, *P. triticina*, and *P. striiformis*, is now very well-established (Garvin, 2011; Ayliffe et al., 2013; Figueroa et al., 2013). Most recently, *B. distachyon* was also recognized as a non-host to *P. emaculata*, the causal agent of switchgrass rust (Gill et al., 2015). Challenging *Brachypodium* spp. with non-adapted rust fungi results in symptoms that are remarkably different than typical symptoms in compatible interactions (Figure 1A; Barbieri et al., 2011; Ayliffe et al., 2013; Figueroa et al., 2013; Gill et al., 2015). The density and appearance of these symptoms varies greatly. Certain *Brachypodium*-rust interactions result in immunity, whereas others can display symptoms at a macroscopic scale, ranging from small necrotic flecks to spreading necrotic and/or chlorotic lesions that may surround small sporulating pustules, responses that are absent in fully-compatible interactions (Figures 1A–E; Ayliffe et al., 2013; Figueroa et al., 2013). In cases where macroscopic symptoms are completely absent due to either failure of the fungus to accomplish plant colonization and/or reach sporulation stages, only microscopic examination of inoculated tissue can allow symptom visualization. *B. distachyon* possesses sufficient chemical, topographical, and thigmotropic signals to induce differentiation of appressoria and in some cases, substomatal vesicles, by most *Puccinia* species examined; although orientation of the germ tube can be affected as in the case of *P. emaculata* (Gill et al., 2015). If fungal growth occurs in the mesophyll space, the infection can advance to the formation of haustoria and sporulating pustules (Figure 2; Ayliffe et al., 2013; Figueroa et al., 2013). Interestingly, the formation of haustoria and pustules was not detected in the *P. emaculata*–*B. distachyon* interaction (Gill et al., 2015). However, such observations could be specific to the *P. emaculata* isolate used in their study. The resistance of *B. distachyon* to *P. graminis* f. sp. *tritici* has been observed as prehaustorial, as most of the infection sites failed to display growth in the plant mesophyll (Figueroa et al., 2013), and post-haustorial, as different races of stem rust fungus can accomplish the formation of colonies that vary in size (Figures 2C–E; Ayliffe et al., 2013). Most importantly for genetic studies, resistance in *Brachypodium* to rust fungi varies substantially across accessions or inbred lines. This phenotypic variability makes it possible to employ genetic-based approaches to map and clone genes governing stem rust resistance in *Brachypodium* species. In fact, differential phenotypes of *B. distachyon* inbred lines Bd3-1 and Bd1-1 in response to *P. brachypodii* allowed the identification of three distinctive rust resistance quantitative trait loci QTLs (Barbieri et al., 2011, 2012). Assessment of the inheritance of the resistance to *P. striiformis* f. sp. *tritici* in two different *B. distachyon* mapping families from parental lines with contrasting phenotypes, BdTR13k × Bd21 and Bd10TR10h × TEK4, suggested that major differences in NHR are simply inherited (Ayliffe et al., 2013). Similar differential responses to *P. graminis* f. sp. *tritici* have been detected in multiple *B. distachyon* inbred lines and accessions (Ayliffe et al., 2013; Figueroa et al., 2013), encouraging comparable resistance inheritance studies against this pathogen.

Our understanding of the genomic architecture controlling NHR in *B. distachyon* is in its infancy. Microscopic analyses and transcriptional profiling have started to shed light into the mechanisms that could mediate resistance in *B. distachyon* against non-adapted rust pathogens. Accumulation of callose and papillae formation has been observed at infection sites during penetration (substomatal vesicles) in interactions with *P. graminis* ff. spp. *tritici*, *lolii*, and *phleipratensis*, *P. striiformis* f. sp. *tritici*, and *P. triticina* (Ayliffe et al., 2013; Figueroa et al., 2013). Interestingly, comparisons of callose deposition at infection sites where the growth of rust fungi was arrested in both host and non-host scenarios, suggests that both types of mechanisms of resistance share common responses and supports the role of PTI in NHR (Ayliffe et al., 2013). Quantification of transcript abundance when three different *B. distachyon* inbred lines were challenged with *P. emaculata* suggest a mixture of responses linked to jasmonic acid, ethylene, and salicylic acid signaling pathways, as well as induction of HRs (Gill et al., 2015). In contrast, evaluation of salicylic acid levels indicated no change in response to *P. graminis* f. sp. *tritici* (Ayliffe et al., 2013). While cell death and dark pigmentation in the rust-infected tissue of various *Brachypodium* accessions and lines is commonly observed (Figures 1C–E; Ayliffe et al., 2013; Figueroa et al., 2013), microscopic analyses indicated that such necrotic responses are not typically associated with autofluorescence, suggesting that hypersensitive cell death is not playing a role in NHR (Ayliffe et al., 2013).

The relative contributions of ETI and PTI to NHR is a long-standing question. It is thought that evolutionary distance between host and non-host plays a major role in determining the relative importance of ETI and PTI processes in these plant–microbe interactions, with ETI favored in non-hosts that are closely related to the natural host (Schulze-Lefert and Panstruga, 2011; Bettgenhaeuser et al., 2014). The phenotypic outcome of *Brachypodium*-rust interactions appear to be highly influenced by the genotype of both plant and fungus, which suggests a relatively strong ETI component to these NHR interactions (Ayliffe et al., 2013; Figueroa et al., 2013; Bettgenhaeuser et al., 2014; Gill et al., 2015). Members of *P. graminis* which are pathogens of plants in the tribe Aveneae and Poeae (ff. spp. *avenae*, *lolii*, *phalaridi*, *phleipratensis*) are more likely to form sporulating pustules on *Brachypodium* accessions than *P. graminis* f. sp. *tritici* (Ayliffe et al., 2013; Figueroa et al., 2013), which supports the view that such interactions resemble more host resistance responses (Ayliffe et al., 2013; Bettgenhaeuser et al., 2014). Future studies are required to carefully evaluate whether ETI is playing a role in the resistance of *B. distachyon* against non-adapted rust pathogens. The mode-of-action of R genes and other broad-spectrum resistance genes is often poorly understood, and therefore, the efficacy of their use in breeding programs is likely undermined. Elucidation of the mechanisms of action of these genes is an area of investigation that could be strengthened by basic research in model plants like *B. distachyon*.

The *B. distachyon* T-DNA insertion mutant resources provide an excellent platform to enable reverse genetics and functional genomic approaches to identify major components of NHR against rust fungi. In addition, the various levels of disease resistance in *Brachypodium* against rust isolates can be exploited to conduct genetic-based approaches to identify, map and clone genes that govern NHR. Recent genomic advances and catalogs of high confidence predicted effectors in North American and Australian stem rust isolates (Duplessis et al., 2011; Upadhyaya et al., 2015) have positioned *P. graminis* f. sp. *tritici* as an ideal organism to investigate the role of ETI during NHR. In combination with the resources available for *B. distachyon*, *P. graminis* f. sp. *tritici* makes a powerful system to investigate the strategies by which obligate biotrophs can be able to reproduce in the face of less suited “host” environments.

Additionally, the *Brachypodium* NHR system could be regarded as a potential source of genetic disease resistance for cereals. The effectiveness of *B. distachyon* derived-genes in transgenic cereals as a strategy for crop protection has not been evaluated yet; however, the high genome collinearity and synteny among the species (International Brachypodium Initiative, 2010) suggests that such genes have high probability of being effective in cereal crops. On the other hand, advances in unraveling the genomic structure and organization of *Hordeum vulgare* (International Barley Genome Sequencing Consortium et al., 2012) and *Triticum aestivum* (Mayer et al., 2014), and the seed transcriptome assembly of *Avena sativa* (Gutierrez-Gonzalez et al., 2013) will enable comparisons with *B. distachyon*, which could be useful to support engineering of disease resistance programs via genome editing techniques.

In addition to its utility in studying NHR, it may be possible to utilize *B. distachyon* for experimentation essentially as a host if sufficiently compatible accession-pathotype combinations can be found. Natural infections of *P. striiformis* resulting in large uredinia have been observed on several accessions grown in nurseries in the Pacific Northwest, where pathotype variation is very high (E. Elmore, Pers. Comm.). Further development of this pathosystem by identifying the most compatible pathotype-accession combination, or possibly generating new lines by crossing the most susceptible accessions, would enable researchers to take advantage of *B. distachyon*’s amenability to transformation. High density marker systems for cereals are enabling candidate R genes to be identified relatively easily, but verification of the candidates are still lacking because transformation systems for wheat and barley are very inefficient. A compatible *B. distachyon* stripe rust system could be used to test candidate stripe rust genes or other components of resistance for function in stably transformed plants. Resistance signaling pathways could be examined by introducing heterologous R genes with, or without, other required donor species components or by using *B. distachyon* mutants. Novel approaches to engineering resistance could be tested in *B. distachyon* transgenics. An example would be RNAi constructs that appear to silence essential rust fungus genes and confer resistance but have only been tested by transient assays to date (Panwar et al., 2013; Yin et al., 2014). Fungal genes, like those encoding putative effectors, could be stably expressed in *B. distachyon* to examine their effects on host physiology to provide insight into their function.

## Conclusions and Remarks

Current trends in food and bioenergy production systems compel the development of transformative approaches to combat plant disease in a sustainable and environmentally safe manner. Rust fungi are among the most important pathogens of grasses and cereals. Understanding the biology of these organisms, especially their mechanisms of virulence and host specificity, is essential to understand emergence of new pathogens or races. Exploring non-host and host pathogen interactions within a single rust species offers unique opportunities to also investigate how new pathogens emerge. Such fundamental knowledge can aid in planning and designing effective and long lasting crop protection strategies. The prospect of exploiting NHR as a natural durable approach to decrease crop yield losses due to biotic stress is well-supported. Defining the molecular and genetic mechanisms conferring NHR is not particularly straightforward given its presumed polygenic nature. However, the amenability of *Brachypodium*-rust fungi as a model pathosystem may accelerate the discovery of molecular and genetic mechanisms underpinning NHR. One avenue to uncover the genetic factors that control disease outcome is the screening of mutagenized *Brachypodium* populations to identify mutants that show susceptibility/compatibility to rust fungi. Combining genetic and genomic tools in both crops and model systems can result in foundational knowledge to support translational plant research. Model-to-crop studies can strengthen plant genetic engineering programs and their potential benefits to agriculture outweigh the time and scale of investments that such studies require. Genome editing techniques and transgenic technologies have quickly emerged as powerful research tools that can enhance traditional plant breeding techniques (Belhaj et al., 2013; Dangl et al., 2013). In spite of regulatory debates and controversial views, these approaches offer great potential to reduce crop yield losses and enable engineering of durable and sustainable plant disease resistance as exemplified by the commercial use of the genetically engineered multiviral resistant squash (Tricoll et al., 1995) and ringspot virus resistant papaya (Lius et al., 1997; Ferreira et al., 2002). Thus, it is worthwhile to invest efforts and research funding to build experimental systems that are meaningful to address immediate societal needs.

### Conflict of Interest Statement

The authors declare that the research was conducted in the absence of any commercial or financial relationships that could be construed as a potential conflict of interest.

## Footnotes

1 http://phytozome.jgi.doe.gov/pz/portal.html

2 http://jgi.doe.gov/our-science/science-programs/plant-genomics/brachypodium/brachypodium-t-dna-collection/

3 http://www.brachytag.org/
